# Correlation between serum laminin level and prognosis of acute heart failure

**DOI:** 10.1002/clc.24056

**Published:** 2023-05-31

**Authors:** Xiaoyun Yan, Jiaqi Ye, Haixiao Chen, Ying Jiang, Ling Xie, Ying Zhang, Wenhui Qiang, Hongli Cai, Koulong Zheng, Qing Zhang

**Affiliations:** ^1^ Department of General Practice Affiliated Hospital 2 of Nantong University Nantong China; ^2^ Department of Cardiology Affiliated Hospital 2 of Nantong University Nantong China

**Keywords:** acute heart failure, major adverse cardiovascular events, prognosis, serum laminin

## Abstract

**Objective:**

To investigate the correlation between serum laminin (LN) level and the prognosis of acute heart failure (AHF).

**Methods:**

A total of 199 patients with AHF treated in Nantong First People's Hospital from March 2019 to November 2021 were included in this study. The patients were divided into the event group and the non‐event group according to whether major adverse cardiovascular events (MACEs) occurred during hospitalization. We collected the baseline data of all patients and their LN levels were measured. The receiver operating characteristic (ROC) curve was used to analyze the predictive value of LN for the occurrence of MACE in AHF patients during hospitalization. Multivariate Logistic regression analysis was used to screen the independent factors associated with the occurrence of MACE in patients with AHF.

**Results:**

Among 199 patients with AHF, 43 were in the event group and 156 were in the non‐event group. The area under ROC curve of LN to predict MACE in AHF patients during hospitalization was 0.8144, 95% confidence interval (CI): 0.7433–0.8855, *p* < .0001, cutoff point = 77.9, specificity 58.33%, and sensitivity 88.37%. Multivariate logistic regression analysis showed that the independent factors associated with the occurrence of MACE in AHF patients were the increase of LN level (odds ratio [OR]: 1.020, 95% CI: 1.012–1.028), the decrease of ejection fraction (OR: 0.007, 95% CI: 0.000–0.362) and diastolic blood pressure (OR: 0.946, 95% CI: 0.913–0.981; *p* < .05).

**Conclusion:**

The increase of LN level is independently correlated with the occurrence of MACE in AHF patients during hospitalization, which has the potential to be a serological indicator for poor prognosis in patients with AHF.

## Graphical abstract

Among 199 patients with acute heart failure (AHF) in our hospital from March 2019 to November 2021, 43 cases developed major adverse cardiovascular events during hospitalization. We found that laminin (LN) levels were significantly elevated in this group of patients (*p* < .001). LN was independently associated with poor prognosis of AHF by receiver operating characteristic curve and multivariate analysis.

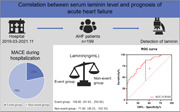

## 1 INTRODUCTION

Compared with chronic heart failure, acute heart failure (AHF) is a group of clinical syndromes with rapid onset or deterioration of symptoms and signs,^1^ and patients are often complicated with organic cardiovascular diseases before onset. This disease can be broadly defined as a “malignant disease” because its high mortality rate exceeds that reported for most cancer diseases.^2^, ^3^

Although the etiology of heart failure is complex, the activation of neurohormones and the increase of inflammatory mediators can be said to be the key factors in the development of ventricular remodeling and heart failure.^4^ Myocardial fibrosis caused by excessive deposition of myocardial extracellular matrix is the main pathological manifestation of ventricular remodeling.^5^ Extracellular matrix is a complex network structure with both strength and plasticity, which is composed of structural proteins and nonstructural proteins, and laminin (LN) is the main component.^6^ However, the current studies on LN and heart failure mostly focus on basic research, and there are few clinical studies on the correlation between serum LN level and the prognosis of AHF. Therefore, the aim of this study was to investigate the correlation between LN and prognosis in patients with AHF.

## 2 MATERIALS AND METHODS

### 2.1 Research objects

**Figure 1:**
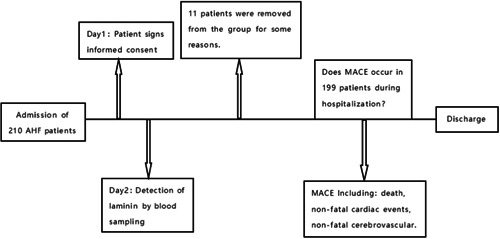
Flow chart.

A total of 210 patients with AHF treated in Nantong First People's Hospital from March 2019 to November 2021 were included in this study. Eleven patients were censored from the analysis due to the following reasons: patients regretting being included in the study, poor patient compliance and withdrawal from the study, and patients lost to follow‐up without access to relevant data. In the end, only 199 people were included in the study. The flow chart is shown in Figure 1. About 199 patients were divided into the event group and the non‐event group according to whether MACE occurred during hospitalization. There were 43 cases in the event group, including 26 males and 17 females, with an average age of 72.44 ± 10.24 years. There were 156 cases in the non‐event group, including 97 males and 59 females, with an average age of 71.65 ± 11.78 years. All patients were diagnosed with AHF according to the European Society of Cardiology guidelines for the diagnosis and treatment of acute and chronic heart failure^7^: 1. Whether there is a history of AHF, suspicious symptoms and signs (such as sitting position, coughing pink foamy sputum, lung wheezing or wet rales); 2. Improve laboratory tests (BNP, blood gases, etc.), electrocardiogram, echocardiography, chest x‐ray, and so forth. AHF can be confirmed if BNP ≥ 100 pg/mL, NT‐proBNP ≥ 300 pg/mL, or MR‐proBNP ≥ 120 pg/mL combined with cardiac ultrasound (the laboratory test index of our hospital is NT‐proBNP). Exclusion criteria: patients with malignant tumors, hematological diseases, autoimmune diseases, rheumatic diseases, chronic respiratory diseases, and severe infections. The study was conducted in accordance with the Declaration of Helsinki (as revised in 2013) and was approved by the Ethics Committee of The Affiliated Hospital 2 of Nantong University, Nantong (IRB: No. 2020KN094). Written informed consent was obtained from each patient. Clinical trials are not applicable to this study and are not registered.

### 2.2 Methods

#### 2.2.1 Collection of baseline data

The basic information of all patients at admission was obtained from medical records, including gender, age, history of diabetes and hypertension, blood pressure, heart rate, liver and kidney function, NT‐proBNP, C‐reactive protein, electrocardiogram, echocardiogram, and so forth.

#### 2.2.2 LN detection

In the morning of the next day after admission, 4 mL of fasting venous blood was collected from all enrolled patients, centrifuged for 10 min at a rate of 2500 r/min. Serum LN levels were determined by chemiluminescence immunoassay (MAGLUMI2000). The kit is provided by Fosun Diagnostic Company. The normal reference range of LN kit in this study is 0.51–50 ng/mL.

#### 2.2.3 Major adverse cardiovascular events

Major adverse cardiovascular events (MACEs) are the most commonly used complex endpoints in cardiovascular studies, including a combination of clinical events: death, nonfatal cardiac events, nonfatal cerebrovascular events, and so forth.^8^

### 2.3 Statistical processing

SPSS25.0 statistical software was used for data analysis. *T* test was used to evaluate continuous variables with normal distribution, expressed as mean ± standard deviation; Chi‐square test was used to evaluate categorical variables; Mann–Whitney *U* test was used to evaluate measurement data with skewed distribution, expressed as median (25%–75%). *p* < .05 was considered statistically significant. Multivariate Logistic regression analysis was used to screen the independent factors associated with MACE in AHF patients during hospitalization. The receiver operating characteristic (ROC) curve was used to evaluate the predictive value of LN for MACE in patients with AHF during hospitalization. GraphPad Prism8 was used for plotting.

## 3 RESULTS

### 3.1 Baseline characteristics

Table 1 summarizes the baseline characteristics of the AHF event group and the non‐event group. Compared with the non‐event group, the levels of NT‐proBNP, LN, urea nitrogen, creatinine, and C‐reactive protein in the event group were significantly increased (*p* < .05). The EF, systolic blood pressure, and diastolic blood pressure levels of patients in the event group were significantly lower than those in the non‐event group (*p* < .05). There were no significant differences in cardiac troponin I (CTNI), gender, age, hypertension, diabetes, heart rate, alanine aminotransferase (ALT), aspartate aminotransferase (AST), total bilirubin, direct bilirubin and indirect bilirubin between the two groups (*p* > .05).

**Table 1:** Comparison of baseline data between the two groups.

	Non‐event group (*n* = 156)	Event group (*n* = 43)	*p*
NT‐proBNP (pg/mL)	5575.50 (3243.25, 10 889.00)	8150.00 (4554.00, 19 000.00)	.010
EF (%)	0.48 ± 0.14	0.41 ± 0.14	.011
CTNI (μg/L)	0.05 (0.01, 1.43)	0.05 (0.02, 6.61)	.742
LN (ng/mL)	71.60 (40.23, 103.10)	159.80 (91.03, 254.50)	.000
Age (years)	71.65 ± 11.78	72.44 ± 10.24	.690
SBP (mmHg)	128.69 ± 21.14	115.07 ± 23.56	.000
DBP (mmHg)	79.92 ± 15.61	69.93 ± 14.18	.000
HR (beats/min)	89.31 ± 23.17	86.53 ± 23.98	.490
ALT (U/L)	23.00 (13.00, 47.00)	20.00 (12.00, 44.00)	.785
AST (U/L)	27.00 (19.00, 58.00)	29.00 (22.00, 43.00)	.588
Tbil (μmol/L)	16.00 (10.90, 23.40)	14.50 (9.70, 25.70)	.751
Dbil (μmol/L)	5.70 (3.60, 8.65)	6.00 (4.40, 12.20)	.102
Ibil (μmol/L)	9.90 (6.90, 13.35)	7.60 (5.00, 14.50)	.076
Cr (μmol/L)	84.25 (70.10, 103.10)	95.30 (80.00, 132.00)	.027
BUN (mmol/L)	6.85 (5.15, 9.52)	8.18 (6.37, 12.96)	.002
CRP (mg/L)	6.72 (3.00, 16.43)	11.27 (3.96, 70.80)	.024
Male, *n* (%)	97 (62.18)	26 (60.47)	.838
Hypertension, *n* (%)	69 (44.23)	13 (30.23)	.099
Diabetes, *n* (%)	42 (26.92)	11 (25.58)	.860

Abbreviations: ALT, alanine aminotransferase; AST, aspartate aminotransferase; BUN, blood urea nitrogen; Cr, creatinine; CRP, C‐reaction protein; CTNI, cardiac troponin I; Dbil, direct bilirubin; DBP, diastolic blood pressure; EF, ejection fraction; HR, heart rate; Ibil, indirect bilirubin; LN, laminin; NT‐proBNP, N‐terminal pro‐brain natriuretic peptide; SBP, systolic blood pressure; Tbil, total bilirubin.

### 3.2 ROC curve

**Figure 2:**
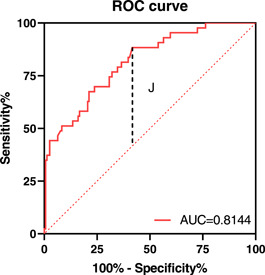
ROC curve. The vertical line J represents the maximum Youden index of 0.467 in this experiment. The corresponding cutoff point is 77.9 ng/mL, the specificity is 58.33%, and the sensitivity is 88.37%. ROC, receiver operating characteristic.

A total of 199 AHF patients were enrolled, including 43 in the MACE event group and 156 in the non‐event group. To explore the effect of LN in predicting the occurrence of MACE in AHF patients during hospitalization, we plotted the ROC curve. The area under the ROC curve is 0.8144, 95% CI: 0.7433–0.8855, *p* < .0001. The Youden index in the ROC curve is also called the correct index. The larger the Youden index, the greater the authenticity. The maximum value of the Youden index corresponds to the optimal diagnostic critical value of the method, which is the cutoff point. In this experiment, the maximum value of the Youden index is 0.467, the corresponding cutoff point is 77.9 ng/mL, the specificity is 58.33%, and the sensitivity is 88.37%. This means that when LN is higher than 77.9 mmol/L, it has a good predictive value for MACE in patients with AHF during hospitalization (see Figure 2).

### 3.3 Multivariate logistic regression analysis

To explore the independent factors associated with MACE in patients with AHF during hospitalization, LN, NT‐proBNP, EF, CTNI, gender, age, presence or absence of hypertension, presence or absence of diabetes, systolic blood pressure, diastolic blood pressure, heart rate, collagen IV, ALT, AST, total bilirubin, direct bilirubin, indirect bilirubin, creatinine, urea nitrogen, and C‐reactive protein were included in binary Logistic regression analysis.

Univariate analysis showed that the increased levels of serum LN, collagen IV, direct bilirubin, urea nitrogen, and C‐reactive protein were significantly positively correlated with MACE (*p* < .05), and the increased levels of EF, systolic blood pressure, and diastolic blood pressure were negatively correlated with MACE (*p* < .05). The above indexes were included in the multivariate logistic regression analysis. The results showed that the independent factors associated with MACE during hospitalization in AHF patients were the increase of LN level (OR: 1.020, 95% CI: 1.012–1.028), the decrease of ejection fraction (OR: 0.007, 95% CI: 0.000–0.362) and diastolic blood pressure (OR: 0.946, 95% CI: 0.913–0.981; *p* < .05, Table 2).

**Table 2:** Logistic regression analysis.

	Univariate					Multivariate			
Index	OR	95% CI		*p*		OR	95% CI		*p*
		Lower limit	Upper limit				Lower limit	Upper limit	
NT‐proBNP (pg/mL)	1.000	1.000	1.000	.687					
EF (%)	0.044	0.004	0.510	.012		0.007	0.000	0.362	.013
CTNI (μg/L)	0.991	0.973	1.009	.326					
LN (ng/mL)	1.018	1.012	1.024	.000		1.020	1.012	1.028	.000
Age (years)	1.006	0.976	1.037	.689					
SBP (mmHg)	0.969	0.952	0.987	.001					
DBP (mmHg)	0.955	0.930	0.980	.000		0.946	0.913	0.981	.003
HR (beats/min)	0.995	0.980	1.010	.488					
ALT (U/L)	1.003	0.998	1.008	.199					
AST (U/L)	1.001	0.999	1.004	.299					
Tbil (μmol/L)	1.018	0.997	1.040	.099					
Dbil (μmol/L)	1.069	1.020	1.120	.005					
Ibil (μmol/L)	0.994	0.952	1.039	.802					
Cr (μmol/L)	1.002	0.998	1.007	.284					
BUN (mmol/L)	1.096	1.027	1.169	.005					
CRP (mg/L)	1.010	1.002	1.018	.019					
Male, *n* (%)	1.075	0.538	2.147	.838					
Hypertension, *n* (%)	1.830	0.888	3.773	.102					
Diabetes, *n* (%)	1.072	0.496	2.317	.860					

Abbreviations: ALT, alanine aminotransferase; AST, aspartate aminotransferase; BUN, blood urea nitrogen; Cr, creatinine; CRP, C‐reaction protein; CTNI, cardiac troponin I; Dbil, direct bilirubin; DBP, diastolic blood pressure; EF, ejection fraction; HR, heart rate; Ibil, indirect bilirubin; LN, laminin; NT‐proBNP, N‐terminal pro‐brain natriuretic peptide; SBP, systolic blood pressure; Tbil, total bilirubin.

## 4 DISCUSSION

AHF refers to a group of clinical syndromes in which acute exacerbation or exacerbation of cardiac dysfunction leads to significant reduction of myocardial contractility and aggravation of cardiac load, resulting in sudden drop in acute cardiac output, sudden increase in pulmonary circulation pressure and increased peripheral circulation resistance.^7^ Although great progress has been made in medical equipment and treatment, the prognosis of patients with AHF is still poor. Its high morbidity and mortality make this disease a major public health problem and a major challenge for current cardiovascular research.

LN was first discovered and named by Timple et al. in 1979. It is a family of heterodimeric trimer glycoproteins composed of one heavy chain and two light chains.^9^ As one of the components of interstitial cells, it is mainly secreted in the hyaline layer of basement membrane, plays the role of adhesion to epithelial cells and matrix, and jointly maintains the network structure of basement membrane with type Ⅲ collagen, thus participating in the process of fibrosis.^10^ In the past, it was common in the study of fibrosis in liver, lung, and other organs. There are few clinical studies on the relationship between LN and heart failure, especially AHF. Therefore, we included 199 patients with AHF to conduct a study related to LN, including 43 patients with MACE during hospitalization and 156 patients in the non‐event group. Multivariate logistic regression analysis showed that LN was independently associated with the occurrence of MACE in AHF patients during hospitalization (OR: 1.020, 95% CI: 1.012–1.028, *p* < .001), indicating poor prognosis of patients with AHF. When LN is higher than 77.9 ng/mL, it has a good predictive value for MACE in AHF patients during hospitalization, the area under the ROC curve is 0.8144, specificity 58.33%, and sensitivity 88.37%. Our study confirmed the correlation between LN and poor prognosis in patients with AHF. The fibrotic cascade involved in LN is a common pathological reaction, which plays a key role in cardiac remodeling and its progression to heart failure.

Previous studies have confirmed that LN levels are elevated in patients with acute myocardial infarction, which leads to ventricular remodeling through cardiac basement membrane remodeling.^11^, ^12^ Evelyn et al. have also shown that basement membrane remodeling plays an important role in myocardial fibrosis in the development of heart failure.^13^ These studies confirm that LN is involved in myocardial fibrosis, and we speculate that the increase of LN reflects a more severe degree of myocardial fibrosis and predicts more adverse cardiovascular events. If this index is included in the existing risk stratification of heart failure, it may be able to predict the adverse outcome of patients early and can be intervened as soon as possible, which can improve the prognosis of patients to some extent. In addition, LN may be used as a therapeutic target for heart failure, changing cardiomyocytes and improving the prognosis of heart failure.^10^

Of course, this study also has some limitations, such as the overall sample size is small, and this study is a cross‐sectional study, although it has been confirmed that there is an independent correlation between LN and poor prognosis of AHF, but large‐scale prospective studies are still needed to further confirm this conclusion.

## CONFLICT OF INTEREST STATEMENT

The authors declare no conflict of interest.

## Data Availability

The data that support the findings of this study are available from the corresponding author upon reasonable request.
